# Exploring the translational disconnect between the murine and human inflammatory response: *in vitro *analysis of the dose-response relationship of LPS and NFκB activation in murine and human immune cells

**DOI:** 10.1186/cc11956

**Published:** 2013-03-19

**Authors:** EP McCarron, I Welters, D Williams, D Antoine, A Kipar

**Affiliations:** 1 University of Liverpool, UK

## Introduction

Inflammation, as seen in sepsis and systemic inflammatory response, is dependent on the activation of the NFκB pathway through Toll-like receptors (TLRs) [1]. Recreating an inflammatory response using lipopolysaccharide (LPS) can provide results that are different to clinical sepsis [2]. By examining NFκB activation in murine and human cells, a species comparison can be made to investigate differences at the cell level that may contribute to the translational disconnect seen *in vivo*.

## Methods

THP1 human monocytes (passages 9 to 11) and RAW 264.7 murine macrophages (passages 15 to 20) were cultured in RPMI-1640 and DMEM respectively and then challenged with LPS. After settling for 24 hours, cells were dosed with six or seven doses of LPS. After 1 hour, nuclear extraction and proteins were separated by acrylamide gel electrophoresis. Membranes where then immunoblotted for actin and p65, followed by densitometric analysis in order to quantify the amount of p65 that had translocated from the cytoplasm to the nucleus (by subtraction from consistent nuclear actin).

## Results

Murine cells required higher doses of LPS compared with human cells in order to detect p65 (human, 1 pg/ml to 100 ng/ml; murine, 30 pg/ml to 1,000 ng/ml). THP1 cells showed a greater fold increase in the p65:actin ratio compared with RAW 264.7 cells. Human cells responded to lower concentrations of LPS. Murine cells appeared to show a molecular resistance to lower doses, but their response was very sensitive at higher doses. A dose-response relationship of LPS dosing and NFκB activation was observed in both cell lines.

## Conclusion

Immunoblotting for p65 is a reliable and reproducible method to determine NFκB activation in cultured cells. Macrophages are more responsive to LPS than monocytes [3] so differences between cell lines would have been expected to be the reverse of what was observed. The species difference in response to LPS may contribute to the apparent disconnect between human and murine responses to LPS and may partially explain the difficulties of translating therapeutic interventions into clinical human sepsis.

## References

[B1] Bonizzi G Karin M Trends Immunol 2004 25 280 288 10.1016/j.it.2004.03.008 15145317

[B2] Remick DG Ward PA Shock 2005 24 Suppl 2 7 11 16374366 10.1097/01.shk.0000191384.34066.85

[B3] Takashiba S Infect Immun 1999 67 11 5573 5578 10531202 10.1128/iai.67.11.5573-5578.1999 PMC96928

